# Jolt accentuation of headache: can this maneuver rule out acute meningitis?

**DOI:** 10.1186/s13104-017-2877-1

**Published:** 2017-10-30

**Authors:** Shirin Afhami, Seyed Ali Dehghan Manshadi, Omid Rezahosseini

**Affiliations:** 1 Department of Infectious Diseases, Shariati Hospital, Tehran University of Medical Sciences, Tehran, Iran; 2 Department of Infectious and Tropical Diseases, Imam Khomeini Hospital Complex, Tehran University of Medical Sciences, Tehran, Iran

**Keywords:** Meningitis, Fever, Headache, Sensitivity and specificity, Predictive value of tests

## Abstract

**Objective:**

Acute meningitis is a medical emergency and its accurate diagnosis could help physicians to accelerate treatment and reduce the mortality and morbidity of patients. Jolt accentuation of headache (Jolt) is an easy clinical maneuver that can be used to diagnose meningitis, but its diagnostic accuracy is controversial. We aimed to assess the “Jolt maneuver” in diagnosis of suspected acute meningitis patients admitted to the emergency ward of Imam-Khomeini Hospital Complex in Tehran, Iran.

**Results:**

Out of 250 patients, 227 were included and 64 (28.2%) had cerebrospinal fluid (CSF) changes compatible with meningitis. Jolt was positive in 40 of 64 (62.5%) meningitis patients. Sensitivity, specificity, positive and negative likelihood ratio (+ LR and − LR) of Jolt were 62.5, 88.3%, 5.36 and 0.42, respectively. These indices were also compared to nuchal rigidity, Kernig’s and Brudzinski’s signs. The highest + LR was for Kernig’s sign (6.79) and the lowest − LR was for nuchal rigidity (0.39). CSF culture was positive in two patients (*Streptococcus pneumoniae* and *Aspergillus* sp.). We found that in adult patients with fever and acute headache, a positive Jolt maneuver has a good diagnostic accuracy for diagnosis of meningitis and indicates a need for CSF assessment, but negative results cannot exclude it.

## Introduction

Among the central nervous system diseases, meningitis is a potentially dangerous and debilitating disease caused by inflammation of the membranes covering the brain and spinal cord. Clinically, meningitis is defined as the presence of meningeal irritation signs (including headache, nuchal rigidity, and loss of consciousness) accompanied by fever for hours to several days. Viruses, bacteria, fungi, and even helminths can cause infection of the central nervous system. Moreover, non-infectious causes such as tumors and collagen vascular diseases can also cause central nervous system involvement and imitate signs of meningeal irritation [1].

When the signs of meningitis and cerebrospinal fluid (CSF) pleocytosis are present but they are not caused by bacterial infection, the condition is called aseptic meningitis [1]. The most common cause of aseptic meningitis is viral infections (85–95% by enteroviruses) and these patients usually recover without treatment [2]. However, bacterial meningitis sometimes has complications even with treatment. These complications include hydrocephalus, cerebral infarction, transitional hernia, and death [3, 4]. Among the bacterial causes, *Haemophilus influenzae*, *Meningococcus*, and *Pneumococcus* comprise a total of approximately 80% of cases [1]. More than 1.2 million people are diagnosed with meningitis annually and this disease is one of the 10 most frequent causes of death worldwide [5, 6]. Therefore, timely diagnosis and treatment of this disease is of particular importance.

Among signs in a case suspicious for meningitis, nuchal rigidity, Kernig’s sign, and Brudzinski’s sign have been noted. In 1882, Vladimir Kernig, who was a Russian neurologist, described his famous Kernig’s sign [7]. A few years later, in 1909, Josef Brudzinski, who was a pediatrician in Poland, reported that among patients with bacterial meningitis or tuberculosis symptoms, Kernig’s sign had a sensitivity of 57% but the sensitivity of Brudzinski’s sign was 96% [8]. Since then, these signs have been used in clinical practice as cardinal signs of meningeal inflammation.

A new maneuver named Jolt accentuation of headache (Jolt) was introduced in 1991 by a group of Japanese researchers for diagnosis of acute meningitis, reporting a sensitivity of 97% and specificity of 60%. Although this was a valuable finding, these results were controversial in other studies based on the strategy of patient selection and type of meningitis [6, 9, 10].

We aimed to assess the frequency and features of Jolt maneuver in patients suspicious for acute meningitis that were admitted to the emergency ward of Imam Khomeini Hospital Complex in Tehran, Iran.

## Main text

### Materials and methods

#### Study design

In this cross-sectional study during May 2015 to September 2016, all patients with the chief complaint of headache and fever that had started in the last 2 weeks were included if they had a diagnosis of acute meningitis. Patients with loss of consciousness or recent head and neck surgery or who did not agree to participate in the study were excluded. Demographic data, including age and sex, duration of headache, and vital signs of the patients were recorded. Results of the laboratory tests including complete blood count and differentiation (CBC/diff), erythrocyte sedimentation rate (ESR), C-reactive protein (CRP), blood sugar, CSF analysis, CSF and blood cultures were extracted from the patient’s documents. In CSF, polymorphonuclears (PMN) were considered predominant if more than 50% of cells were neutrophils. A single physician performed all of the physical examinations before evaluating the results of the laboratory tests. The study was approved by the ethics committee of the Tehran University of Medical Sciences (No. IR.TUMS.REC. 1394.1620).

The gold standard for a diagnosis of meningitis is defined as a CSF white blood cell count (WBC) higher than 5 with or without a smear and/or culture positive cerebrospinal fluid; otherwise, it is considered negative as mentioned in previous studies [6, 10, 11].

#### Physical examinations

All of the physical examinations were done in a supine and comfortable position. We did not prescribe analgesic drugs to patients before examination. In cases of nuchal rigidity, the head of the patient was flexed by the examiner toward the chest and if there was rigidity in the neck movement the test was considered positive [4, 7]. Kernig’s sign was positive if the patient had back pain or resistance during the extension of a 90° flexed hip and knee [4, 7]. For Brudzinski’s sign, while the legs were extended, the neck was bent towards the chest and in the event of involuntary flexion of the knee and hip, a positive test was reported [4, 7]. The Jolt maneuver was positive if the patient with extended feet had an exacerbation of headache when the head was rotated with a frequency of 2–3 times per second in the horizontal axis.

In patients with focal neurologic deficits or suspected increased intracranial pressure as well as patients with a history of seizures, a brain computed tomography scan (CT scan) was performed.

#### Statistics and data analysis

According to a study conducted on 14 patients, the frequency of a positive Jolt maneuver in patients with fever, headache, and neck stiffness was 63% [11]. Considering P = 0.63 according to the Cochrane formula, z = 1.96, and d = 0.063, the required sample size was approximately 226 people and after including 10% lost to follow-up, a sample size of 250 patients was considered. Descriptive statistics, Chi square, and independent-sample t-tests (sig. 2-tailed) were used for data analysis. Sensitivity, specificity, positive and negative predictive values (PPV and NPV), and positive and negative likelihood ratios (+ LR and − LR) were calculated. P ≤ 0.05 was considered statistically significant. SPSS software (version 19, SPSS Inc., Chicago, IL, USA) was used for data analysis.

### Results

A total of 250 patients were assessed and 227 patients were included in the final statistical analysis. Of these, 98 (43.2%) were women and 129 (56.8%) were men (Chi square test P = 0.480). Their mean age was 46.5 ± 17.63 (range 18–80) years.

CSF analysis diagnosed meningitis in 64 (30 women/34 men) patients. The Jolt maneuver was positive in 40/64 (62.5%) of meningitis and 19/163 (11.7%) of non-meningitis patients (Chi square test P < 0.001).

Based on the positive or negative Jolt maneuver status (J-pos and J-neg, respectively), meningitis patients were divided into two groups and clinical findings and CSF characteristics were compared (an independent t-test was applied). The results of this comparison are shown in Table 1.

**Table 1:** Comparison of clinical findings and CSF characteristics in meningitis patients based on Jolt maneuver status

Variable	Jolt	Frequency	Mean ± SD	t	Df	Sig. (2-tailed)*
Age (years)	–	24	43.25 ± 16.05	0.142	62	0.887
	+	40	42.62 ± 17.59			
Duration of headache (days)	–	24	4.08 ± 3.39	0.749	60	0.457
	+	38	3.55 ± 2.18			
Body temperature	–	23	38.24 ± 0.33	− 1.651	60.63	0.104
	+	40	38.43 ± 0.54			
RR/min	–	19	18.05 ± 2.34	− 2.220	52	0.031
	+	35	19.51 ± 2.29			
PR/min	–	21	85.04 ± 11.59	− 2.639	58	0.011
	+	39	91.97 ± 8.53			
Sys BP (mmHg)	–	23	118.34 ± 18.86	0.188	61	0.85
	+	40	117.57 ± 16.12			
Dias. BP (mmHg)	–	23	74.39 ± 9.99	− 0.020	61	0.984
	+	40	74.45 ± 12.08			
WBC (cell/µl)	–	17	9184 ± 4180	− 2.236	41	0.031
	+	26	11721 ± 3244			
CRP	–	17	53.98 ± 59.08	− 0.193	37	0.848
	+	22	57.16 ± 43.74			
ESR (mm)	–	19	26.47 ± 24.45	0.680	38	0.501
	+	21	32.09 ± 27.49			
Sugar (mg/dl) CSF	–	24	53.37 ± 18.99	0.07	61	0.938
	+	39	52.74 ± 36.28			
Protein (mg/dl)	–	24	65.81 ± 43.46	− 0.79	61	0.431
	+	39	73.90 ± 36.54			
LDH	–	24	78.08 ± 86.30	− 1.38	55.54	0.171
	+	37	125.28 ± 177.03			
WBC (cell/µl)	–	24	276.54 ± 471.71	− 1.01	62	0.312
	+	40	1338.55 ± 5074.41			
PMN (cell/µl)	–	24	60.16 ± 97.93	− 2.32	38.17	0.025
	+	38	431.68 ± 975.97			

* Independent sample *t*-test

When grouped by the percentage of PMN cells in the CSF, the Jolt maneuver was positive in 23/29 (79.31%) of PMN dominant patients and 17/35 (48.6%) of lymphocyte-dominant patients (Chi square test P = 0.011). The frequency and percentage of evaluated signs on physical examinations according to the type of meningitis are shown in Table 2.

**Table 2:** Frequency (%) of evaluated signs in physical examinations according to type of meningitis

Clinical sign	Type of meningitis		P-value (2-sided)*
	PMN dominant (N = 29)	Lymphocyte dominant (N = 35)	
	n (%)	n (%)	
Jolt accentuation	23 (79.3)	17 (48.6)	0.011
Nuchal rigidity^a^	21 (75.0)	28 (80.0)	0.635
Kering’s sign	14 (43.8)	18 (56.3)	0.802
Brudzinski’s sign	20 (69.0)	21 (60.0)	0.457

* Chi square test

^a^Measured in 226 patients

The sensitivity and specificity of the Jolt maneuver for the diagnosis of meningitis was 62.5 and 85.7%, respectively, and the PPV was 67.7%, the NPV was 85.7%, and the + LR and − LR were 5.36 and 0.42, respectively.

Among the patients with meningitis, the CSF smear was negative in all of the samples. The CSF culture was positive for *S. pneumoniae* and *Aspergillus* sp. in two samples. Blood cultures were available for 40 patients with meningitis and only one patient had a positive blood culture for *S. pneumoniae*. In non-meningitis patients, the blood culture was positive in 10 patients. Micro-organisms such as *Alcaligenes* sp., *Klebsiella oxytoca, S. epidermidis*, and *Stenotrophomonas maltophilia* were considered to be due to contamination and excluded from the results.

### Discussion

The Jolt maneuver in patients with meningitis has been previously reported to have a high sensitivity for the detection of acute meningitis. However, subsequent studies challenged the results of the initial study and it was found that many factors positively or negatively affected this maneuver.

In our study, at total of 227 patients were enrolled, and 64 patients (28.2%) had meningitis; no significant difference was found between age and gender of meningitis and non-meningitis patients. The frequencies of positive Kernig’s sign, Brudzinski’s sign, nuchal rigidity, and the Jolt maneuver in patients with meningitis were significantly higher than among non-meningitis patients. The most common sign in examinations of patients with meningitis (77.8%) and non-meningitis (43.6%) was nuchal rigidity (P < 0.001). This finding is similar to results of a study by Aminzadeh and colleagues (78.6%) [11].

The mean of the CSF-PMN count was significantly higher in J-Pos patients (P = 0.025) but this factor was not measured in previous studies. Hence, we cannot perform a comparison [6, 7, 10–12].

The sensitivity and specificity of the Jolt maneuver in the diagnosis of meningitis were 62.5 and 85.7%, respectively, and the PPV, NPV, + LR, and − LR were 67.7, 85.7%, 5.36, and 0.42, respectively. These findings indicate that, although a positive Jolt maneuver strongly increases the possibility of meningitis, a negative result is not able to rule out the presence of acute meningitis.

Uchihara first evaluated the Jolt maneuver in patients with meningitis in 1991 [10]. The sensitivity, specificity, + LR, and − LR of the Jolt in Uchihara’s study were 97.1, 60%, 4.2, and 0, respectively. That study had a small sample size and was performed at a time when the incidence of viral meningitis was high. We performed our study with a larger sample size and a longer study period to reduce any effect of these confounding factors.

We compared our results (sensitivity, specificity, PPV and NPV, + LR and − LR) with previous studies in Table 3. Most of the previous studies, including a recent study by Sato and colleagues [13], were performed retrospectively and the examiners were all different, but we performed a prospective study and only one physician did all of the examinations to reduce bias.

**Table 3:** Sensitivity, specificity, PPV, NPV, + LR and − LR of evaluated signs in compare to previous studies

Author	Study population	Micro-organism (frequency)	Sign/maneuver	Sensitivity %	Specifity %	PPV	NPV	LR +	LR −
				(0.95 CI)	(0.95 CI)	(0.95 CI)	(0.95 CI)		
Uchihara [9]^a^	54	Viral (28) Bacterial (1) Tuberculosis (1)	Jolt accentuation	97.1	60	80.5	92.3	4.2	0.0
Thomas et al. [8]^b^	297	Viral (9) Bacterial (3) Fungal (6)	Jolt accentuation	NR	NR	NR	NR	NR	NR
			Nuchal rigidity	30	68	26	73	0.94	1.02
			Kernig’s sign	5	95	27	72	0.97	1
			Brudzinski sign	5	95	27	72	0.97	1
Aminzadeh et al. [11]^a^	14	Undefined	Jolt accentuation	100	71.5	78	100	1	0
			Nuchal rigidity	100	57	70	100	2.33	0
Waghdhare et al. [6]^a^	190	Viral (62) Bacterial (7) Tuberculosis (30)	Jolt accentuation	6.06 (2.26–12.7)	98.9 (94–100)	NR	NR	5.52	0.95
			Nuchal rigidity	39.4 (29.7–49.7)	70.3 (59.8–79.5)	NR	NR	1.33	0.86
			Kernig’s sign	14.1 (7.95–22.6)	92.3 (84.8–96.9)	NR	NR	1.84	0.93
			Brudzinski sign	11.1 (5.68–19)	93.4 (86.2–97.5)	NR	NR	1.69	0.95
Tamune et al. [12]^c,d^	113	Viral (38)	Jolt accentuation	78.9 (66.0–91.9)	32.0 (21.4–42.6)	NR	NR	1.16	0.66
Nakao et al. [7]^a^	230	Viral (1) Bacterial (1) Fungal (1)	Jolt accentuation	21	82	NR	NR	1.2	1
			Nuchal rigidity	13	80	NR	NR	0.6	1.1
			Kernig’s sign	2	97	NR	NR	0.8	1
			Brudzinski sign	2	98	NR	NR	1	1
Sato et al. [13]^a,e^	108	Undefined	Jolt accentuation	75 (61.8–84.8)	35.1 (24–48)	NR	NR	1.16	0.71
This study	227	Bacterial (1) Fungal (1)	Jolt accentuation	62.5 (49.4–74)	88.3 (82.1–92.6)	67.7 (54.2–79)	85.7 (79.2–90.4)	5.36	0.42
			Nuchal rigidity	77.7 (65.2–86.9)	56.4 (48.4–64.1)	40.8 (32–50.1)	86.7 (78.5–92.3)	1.78	0.39
			Kernig’s sign	50 (37.362.6)	92.6 (87.2–95.9)	72.7 (56.9–84.5)	82.5 (76–87.5)	6.79	0.53
			Brudzinski sign	64 (51.3–75.3)	74.2 (66.6–80.6)	49.3 (38.3–60.5)	84 (76.7–89.4)	2.48	0.48

*NR* no result

^a^Meningitis was defined as WBC > 5 cell/µl

^b^Meningitis was defined as WBC > 6 cell/µl

^c^Meningitis was defined as WBC > 15.3 cell/µl

^d^Study population was 193 patients and 113 had fever and headache without loss of consciousness

^e^Conscious patients

As mentioned in previous studies, the sensitivity of the Jolt maneuver for detection of granulomatous meningitis, such as tuberculosis meningitis, is low [6]. Therefore, decision-making solely based on this maneuver in countries with a high TB prevalence will result in missing a significant number of TB meningitis patients. The inclusion criteria and the frequency of meningitis in each study population will affect the diagnostic sensitivity of the Jolt maneuver. This issue was noted in an article published in 2014 [9].

Jolt accentuation of headache can occur in some other intracranial pathologies. For example, in patients with migraine, the headache exacerbates during the Jolt maneuver, and also 67% of intracranial space occupying lesions have positive Jolt maneuver results [14]. The use of analgesics and carcinomatous meningitis can also affect the results of the Jolt maneuver [10, 15]. Our patients were febrile and did not have any focal neurologic deficits or known cancers and we excluded intracranial lesions by imaging. We did not prescribe analgesic drugs to our patients; however, some patients consumed oral painkillers such as acetaminophen or non-steroidal anti-inflammatory drugs (NSAIDs) before their admission.

One of our patients had bacterial and one had a fungal-positive CSF culture. Thomas and colleagues had three bacterial and six fungal positive CSF cultures [8], Whaghdhare had 3 bacterial but no fungal positives [6], and Nakao had 1 bacterial and 1 fungal positive CSF cultures [7]. Therefore, we can claim that we have good accuracy in finding cases with positive CSF cultures. However, owing to lack of samples for routine polymerase chain reaction (PCR) examination of CSF, we were unable to detect any viruses.

In conclusion, in adult patients with fever and acute headache, a positive Jolt maneuver has a good diagnostic accuracy for diagnosis of meningitis and indicates a need for assessment of the CSF, although negative Jolt results cannot exclude meningitis and the final clinical judgment rests with the physician.

### Limitations

Although our prospective study with an appropriate sample size, and examination of all patients by a single physician had many strengths, we also had some limitations including the lack of routine testing for tuberculosis and viruses on CSF and the impossibility of a final diagnosis for all patients who presented with fever and headache but did not have meningitis, and therefore, we cannot estimate the false positive and false negative rates of the Jolt maneuver.

## Abbreviations

Jolt: Jolt accentuation of headache
CSF: cerebrospinal fluid
CBC: complete blood count
PMN: polymorphoneuclear
ESR: erythrocyte sedimentation rate
CRP: C-reactive protein
NSAIDs: non-steroidal anti-inflamatory drugs
PCR: polymerase chain reaction
+ LR: positive likelihood ratio
− LR: negative likelihood ratio
PPV: positive predictive values
NPV: negative predictive values
J-pos: positive Jolt maneuver
J-neg: negative Jolt maneuver
CT scan: computed tomography scan
